# Supplementary material for: Sodium Intake and Proteinuria/Albuminuria in the Population—Observational, Cross-Sectional Study
Source: Nutrients. 2021 Apr 11;13(4):1255. doi: 10.3390/nu13041255 (PMC8068813; doi:10.3390/nu13041255)
Supplement: Supplementary file 1 [file nutrients-13-01255-s001.pdf]

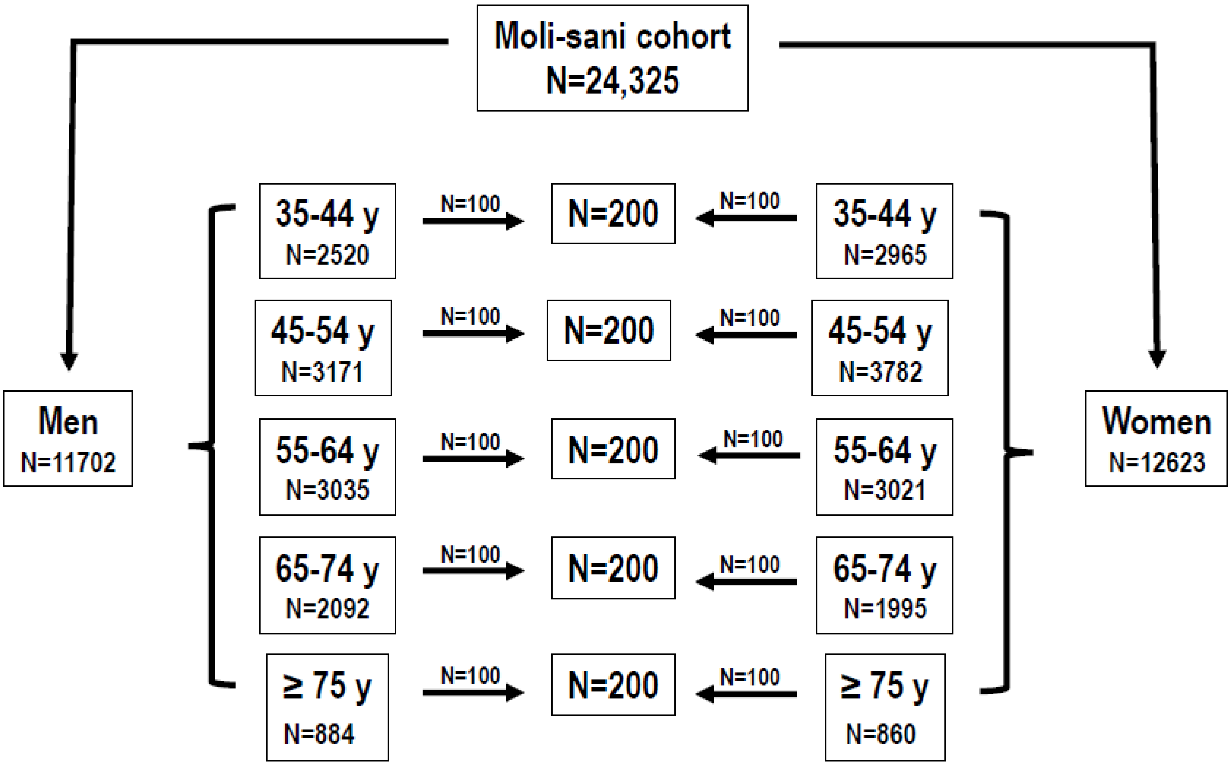

Figure S1. Selection of the study population from Moli-sani cohort.

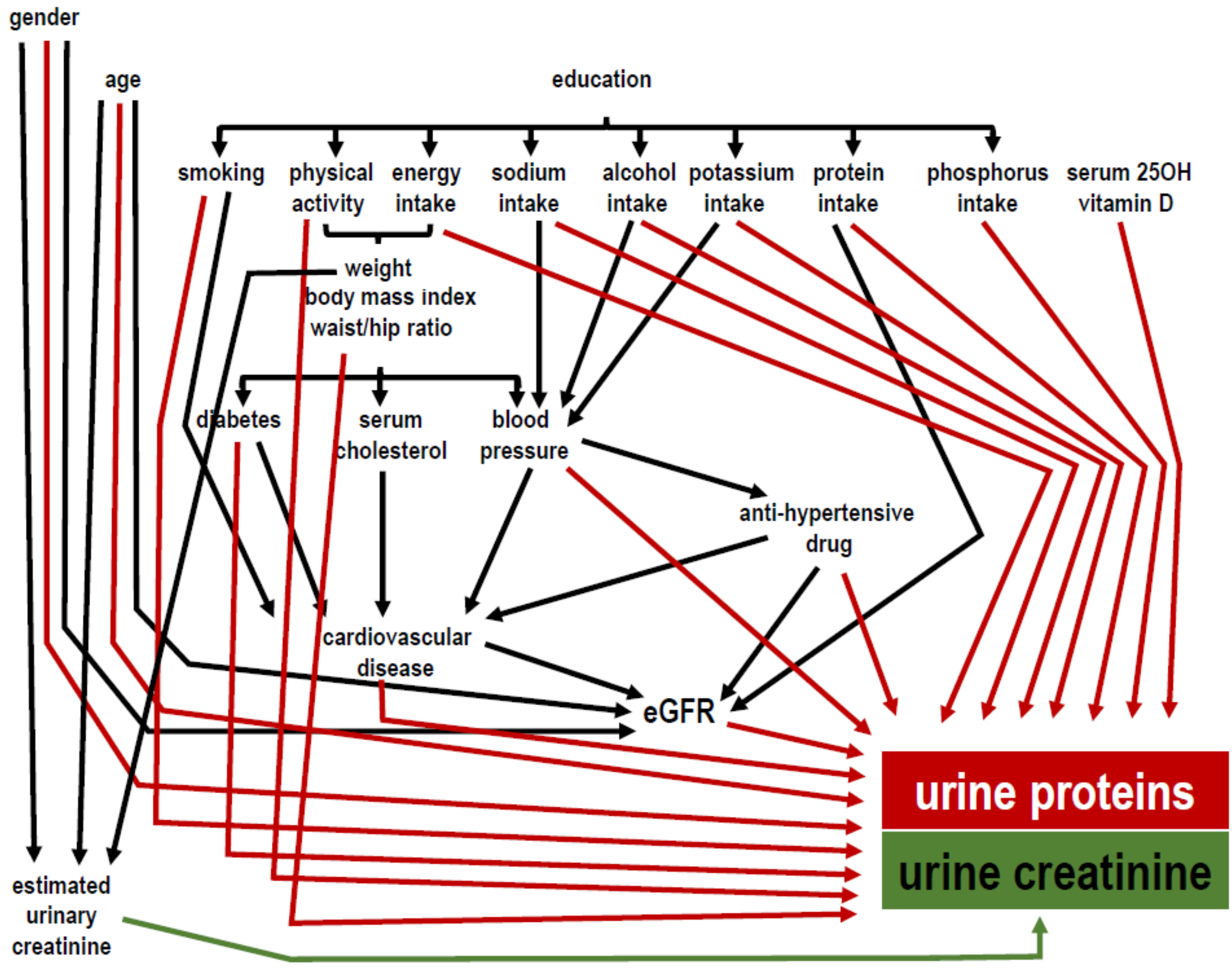

Figure S2. Directed Acyclic Graph (DAG) for justification of the exclusion of education, serum cholesterol, and statin from the multi-variable analyses.

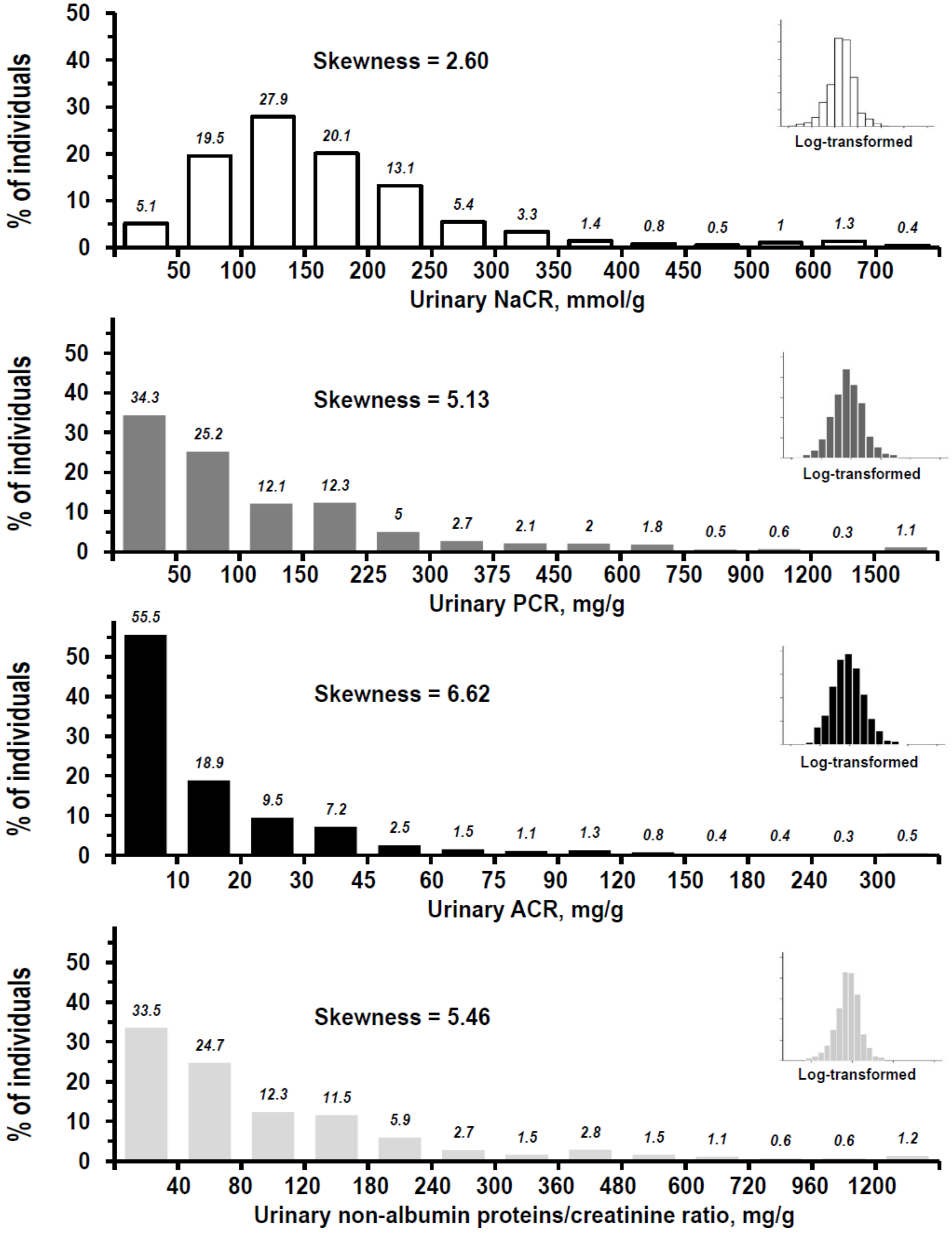

Figure S3. From top to bottom panel: frequency distribution and skewness of urinary NaCR (open bars), urinary PCR (dark grey bars), urinary ACR (black bars), and urinary non-albumin proteins to creatinine ratio (light grey bars). For each panel, the inserts in the top right corner shows the frequency distribution of log-transformed data.

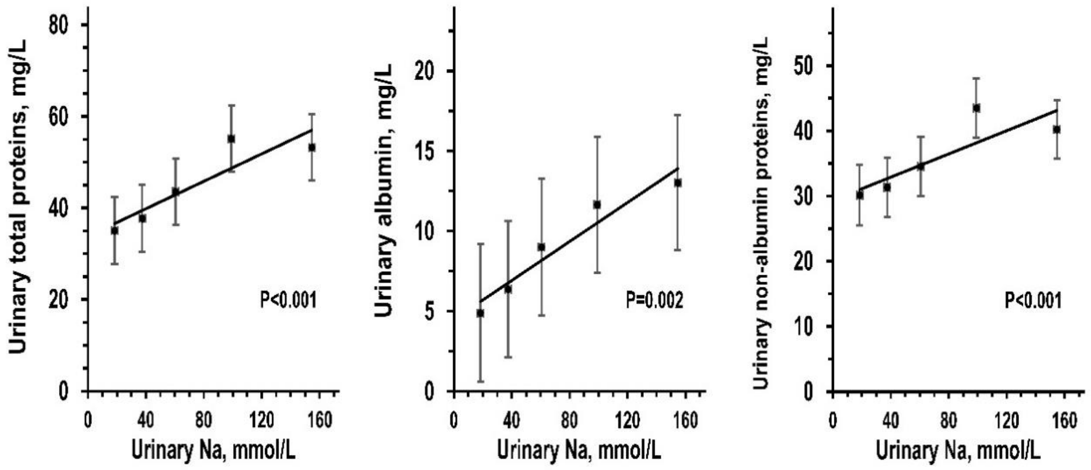

Figure S4. Multi-variable ANOVA by quintile of non-transformed urine sodium concentration as mmol/L for non-transformed urine concentrations of total proteins (left panel), albumin (central panel), and non-albumin proteins (right panel): mean with 95%CI. Number of individuals from quintile 1 to quintile 5 = 186, 188, 188, 188, and 186. ANOVAs were controlled for the following co-variates: age, body mass index, waist/hip ratio, estimated urinary creatinine excretion, smoking, systolic pressure, diastolic pressure, diabetes, history of cardiovascular disease, eGFR, calorie intake, reported treatment with antihypertensive drug, inhibitor or blocker of the renin-angiotensin system, diuretic, and log-transformed data of total physical activity, leisure physical activity, alcohol intake, urinary ratios of urea nitrogen to creatinine, potassium to creatinine, phosphorus to creatinine, and serum total 25(OH) vitamin D.

Table S1. Multi-variable logistic regression analyses of high urinary PCR, of high urinary ACR, and of high urinary non-albumin proteins to creatinine ratio alternatively regressed over NaCR quintiles: odds ratio (OR) with 95%CI for difference between two consecutive quintiles in the whole study cohort and in selected subgroups. Tabular presentation of data shown in Figure 2.

	Number of individuals	Dependent variable								
		urinary PCR ≥ 150 mg/g			urinary ACR ≥ 30 mg/g			urinary non/albumin proteins ≥ 120 mg/g		
		OR	95%CI	P	OR	95%CI	P	OR	95%CI	P
All	936	1.81	1.55/2.12	<0.001	1.62	1.35/1.95	<0.001	1.84	1.58/2.16	<0.001
Men	482	1.85	1.45/2.35	<0.001	1.54	1.15/2.05	0.003	1.91	1.50/2.42	<0.001
Women	454	1.89	1.50/2.37	<0.001	1.70	1.32/2.19	<0.001	1.89	1.51/2.38	<0.001
Age ≥ 65 years	186	2.46	1.59/3.81	<0.001	2.65	1.61/4.37	<0.001	2.58	1.64/4.06	<0.001
Age < 65 years	750	1.77	1.48/2.11	<0.001	1.53	1.24/1.89	<0.001	1.79	1.50/2.13	<0.001
eGFR < 90 mL/min x 1.73 m^2	332	1.70	1.41/2.06	<0.001	1.47	1.19/1.82	<0.001	1.83	1.51/2.23	<0.001
eGFR ≥ 90 mL/min x 1.73 m^2	604	2.26	1.64/3.11	<0.001	2.19	1.45/3.31	<0.001	1.96	1.46/2.62	<0.001
Obese	329	1.62	1.24/2.13	0.001	1.51	1.11/2.06	0.009	1.65	1.26/2.16	<0.001
Non-obese	607	1.95	1.60/2.39	<0.001	1.80	1.40/2.30	<0.001	1.99	1.63/2.43	<0.001
Drinker	667	1.76	1.46/2.13	<0.001	1.49	1.19/1.85	<0.001	1.86	1.54/2.25	<0.001
Non-drinker	269	2.14	1.56/2.92	<0.001	1.97	1.37/2.85	<0.001	2.07	1.52/2.82	<0.001
Smoker	205	1.98	1.35/2.93	<0.001	1.67	1.01/2.75	0.044	2.63	1.68/4.11	<0.001
Non-smoker	731	1.86	1.55/2.23	<0.001	1.65	1.35/2.03	<0.001	1.83	1.53/2.18	<0.001
Hypertensive	650	1.67	1.40/2.00	<0.001	1.62	1.32/1.99	<0.001	1.71	1.43/2.05	<0.001
Non-hypertensive	286	2.90	1.95/4.31	<0.001	1.73	1.05/2.84	0.032	2.67	1.85/3.86	<0.001
With hypercholesterolemia	302	1.71	1.31/2.24	<0.001	1.76	1.26/2.45	0.001	1.66	1.27/2.16	<0.001
Without hypercholesterolemia	634	1.89	1.55/2.31	<0.001	1.62	1.28/2.04	<0.001	2.02	1.65/2.48	<0.001
Diabetic	120	2.78	1.61/4.80	<0.001	1.52	0.96/2.41	0.077	4.76	2.15/10.54	<0.001
Nondiabetic	816	1.73	1.46/2.05	<0.001	1.76	1.42/2.18	<0.001	1.71	1.45/2.02	<0.001
With cardiovascular disease	60	1.21	0.33/4.40	0.778	not calculable			not calculable		
Without cardiovascular disease	876	1.87	1.59/2.20	<0.001	1.67	1.38/2.02	<0.001	1.87	1.59/2.20	<0.001

Analyses were controlled for the following co-variates: age, body mass index, waist/hip ratio, estimated urinary creatinine excretion, smoking, systolic pressure, diastolic pressure, diabetes, history of cardiovascular disease, eGFR, calorie intake, reported treatment with antihypertensive drug, inhibitor or blocker of the renin-angiotensin system, diuretic, and log-transformed data of total physical activity, leisure physical activity, alcohol intake, urinary ratios of urea nitrogen to creatinine, potassium to creatinine, phosphorus to creatinine, and serum total 25(OH) vitamin D.

The Moli-sani research group

Steering Committee: Licia Iacoviello* (Chairperson), Giovanni de Gaetano* and Maria Benedetta Donati*. Scientific secretariat: Marialaura Bonaccio*, Americo Bonanni*, Chiara Cerletti*, Simona Costanzo*, Amalia De Curtis*, Augusto Di Castelnuovo§, Francesco Gianfagna°§, Mariarosaria Persichillo*, Teresa Di Prospero* (Secretary). Safety and Ethical Committee: Jos Vermylen (Catholic Univesity, Leuven, Belgio) (Chairperson), Ignacio De Paula Carrasco (Accademia Pontificia Pro Vita, Roma, Italy), Antonio Spagnuolo (Catholic University, Roma, Italy).

External Event adjudicating Committee: Deodato Assanelli (Brescia, Italy), Vincenzo Centritto (Campobasso, Italy). Baseline and Follow-up data management: Simona Costanzo* (Coordinator), Marco Olivieri (Associazione Cuore Sano, Campobasso, Italy), Teresa Panzera*. Data Analysis: Augusto Di Castelnuovo§ (Coordinator), Marialaura Bonaccio*, Simona Costanzo*, Simona Esposito*, Alessandro Gialluisi*, Francesco Gianfagna°§, Emilia Ruggiero*. Biobank and biochemical laboratory: Amalia De Curtis* (Coordinator), Sara Magnacca§. Genetic laboratory: Benedetta Izzi* (Coordinator), Annalisa Marotta*, Fabrizia Noro*, Roberta Parisi*, Alfonsina Tirozzi*. Recruitment staff: Mariarosaria Persichillo* (Coordinator), Francesca Bracone*, Francesca De Lucia (Associazione Cuore Sano, Campobasso, Italy), Cristiana Mignogna°, Teresa Panzera*, Livia Rago*. Communication and Press Office: Americo Bonanni*. Regional Health Institutions: Direzione Generale per la Salute - Regione Molise; Azienda Sanitaria Regionale del Molise (ASReM, Italy); Molise Dati Spa (Campobasso, Italy); Offices of vital statistics of the Molise region. Hospitals: Presidi Ospedalieri ASReM: Ospedale A. Cardarelli – Campobasso, Ospedale F. Veneziale – Isernia, Ospedale San Timoteo - Termoli (CB), Ospedale Ss. Rosario - Venafro (IS), Ospedale Vietri – Larino (CB), Ospedale San Francesco Caracciolo - Agnone (IS); Casa di Cura Villa Maria - Campobasso; Ospedale Gemelli Molise - Campobasso; IRCCS Neuromed - Pozzilli (IS). *Department of Epidemiology and Prevention, IRCCS Neuromed, Pozzilli, Italy - °Department of Medicine and Surgery, University of Insubria, Varese, Italy

§Mediterranea Cardiocentro, Napoli, Italy. Baseline Recruitment staff is available at https://www.moli-sani.org/?page_id=173Decreto 1588 and from the Instrumentation Laboratory (Milan, Italy).
